# Supplementary material for: The Contribution of Germline Pathogenic Variants in Breast Cancer Genes to Contralateral Breast Cancer Risk in BRCA1/BRCA2/PALB2-Negative Women
Source: Cancers (Basel). 2023 Jan 8;15(2):415. doi: 10.3390/cancers15020415 (PMC9856968; doi:10.3390/cancers15020415)
Supplement: Supplementary file 1 [file cancers-15-00415-s001.zip › Larionov_WECARE_Supp_Figures_07Jan2023.pdf]

Supplementary figures

The contribution of germline pathogenic variants in breast cancer genes to contralateral breast cancer in *BRCA1/BRCA2/PALB2*-negative women

A. Larionov, E. Fewings, J. Redman, M. Goldgraben, G. Clark, J. Boice, P. Concannon, J. Bernstein, D.V. Conti, the WECARE Study Collaborative Group, M. Tischkowitz

Supp. Figure S1: Study flowchart ***2***

Supp. Figure S2: PCA for Group 1 .... ***3***

A: Projecting to 1KGP **3**

B: Scree plot **3**

Supp. Figure S3: PCA for Group 2 .... ***4***

A: Projecting to 1KGP **4**

B: Scree plot **4**

Supp. Figure S4: WECARE-Group1 + NFFE PCA Scree plot ***5***

Supp. Figure S5: Burden of PGVs stratified by latency ***6***

A: Group 1 .... **6**

B: Group 2 .... **6**

Supp. Figure S6: WECARE-Group1 + NFFE PGVs burden ***7***

Supp. Figure S1: Study flowchart

Secondary bioinformatics
following Broad’s Best Practices
- FastQC and FASTQ Trimming
- Alignment (BWA MEM, GRCh37)
- BAM files QC and processing
- Variant Calling (GATK HC)
- Joined genotyping with NFFE
- Variants QC and filtering (VQSR & hard filters)
- Variants normalization & splitting multiallelic sites
- Variants Annotation (VEP, ClinVar, CADD)

Samples check and filtering
- pairwise concordance
- concordance with previous GWAS data

PCA
- PC-s for regression models,
- ethnicity assessment by projecting to 1KGP

Statistical analysis
- crude counts for bar-plots
- regression models for statistical significance

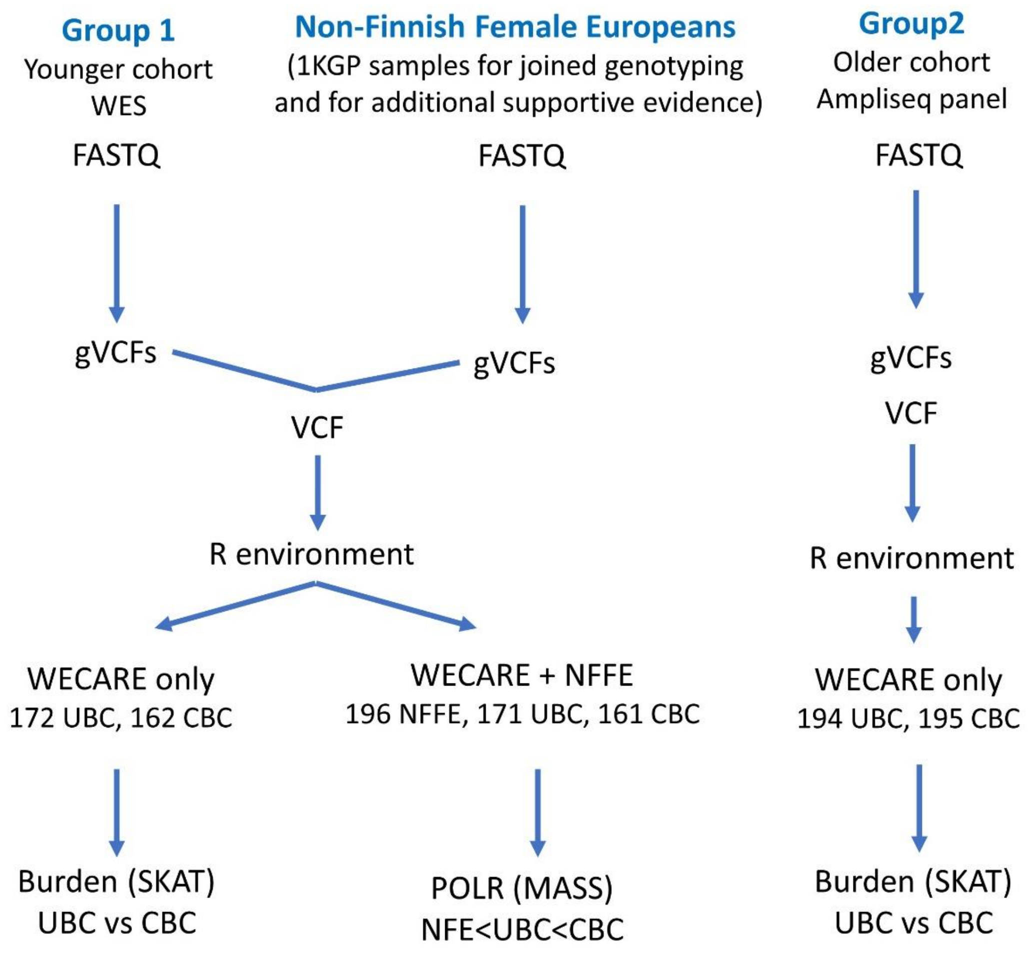

Supp. Figure S2: PCA for Group 1

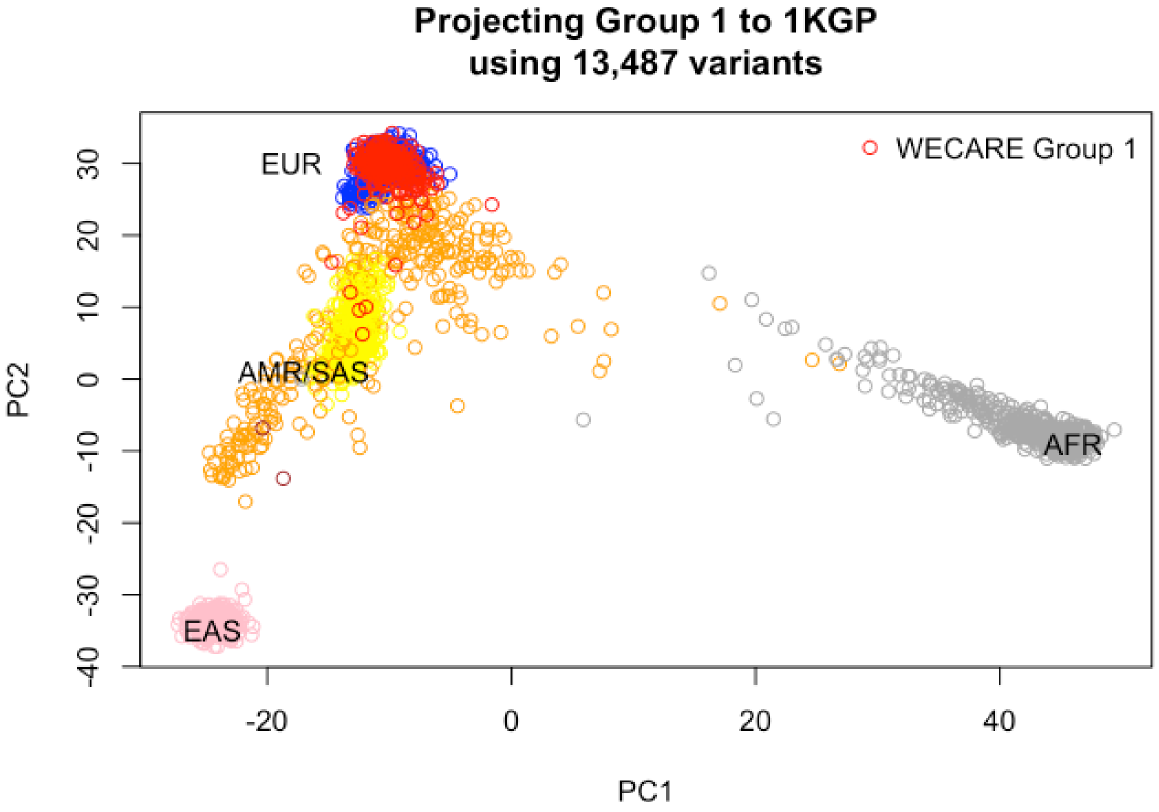

B: Scree plot

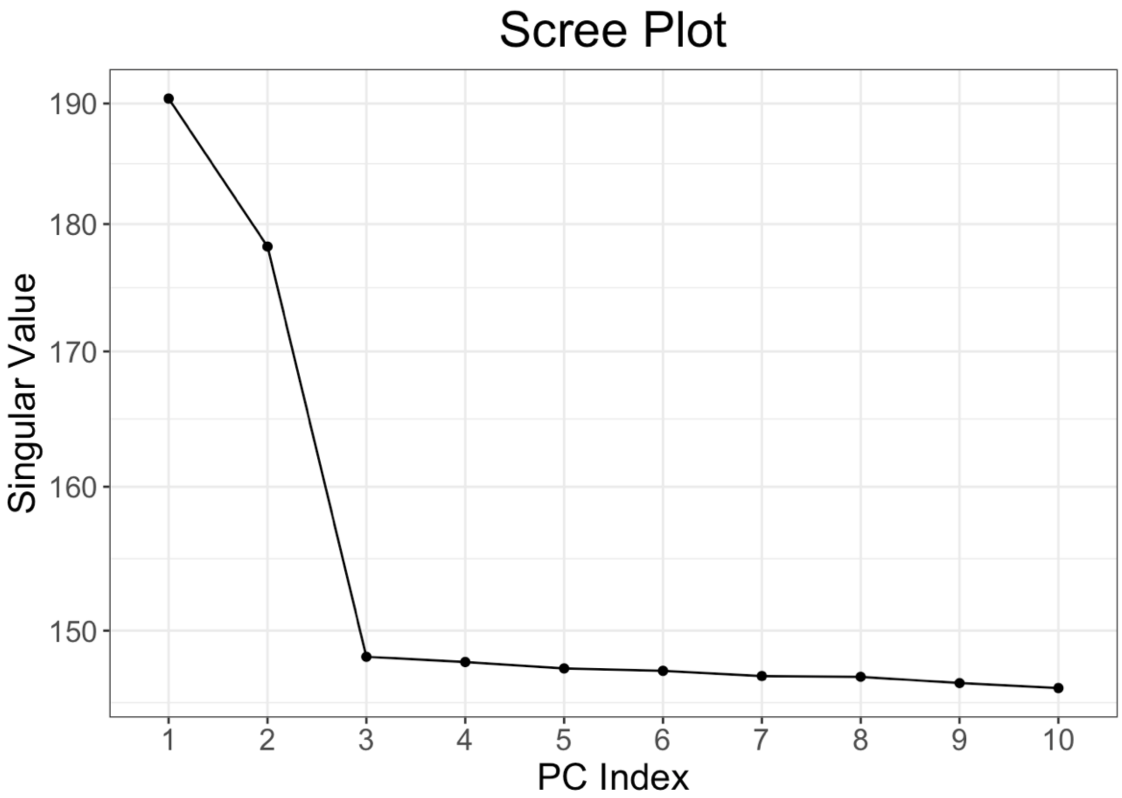

Supp. Figure S3: PCA for Group 2

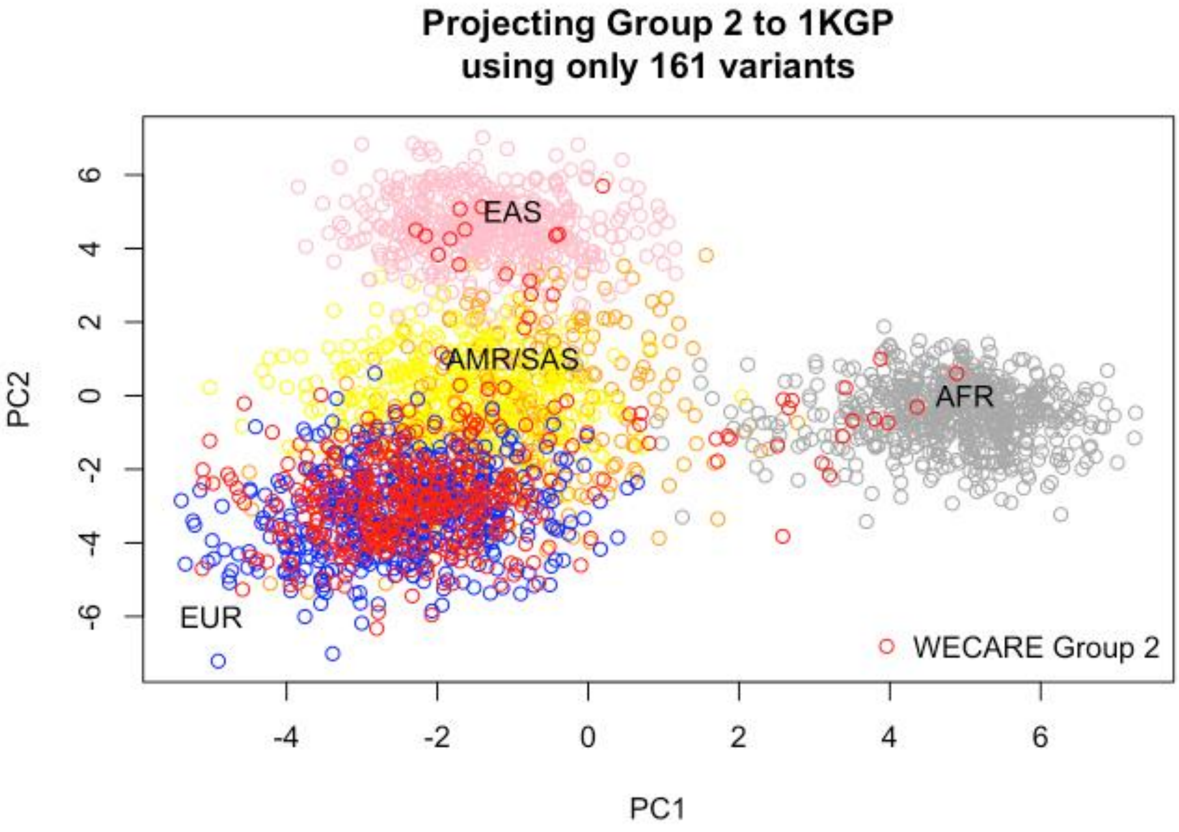

B: Scree plot

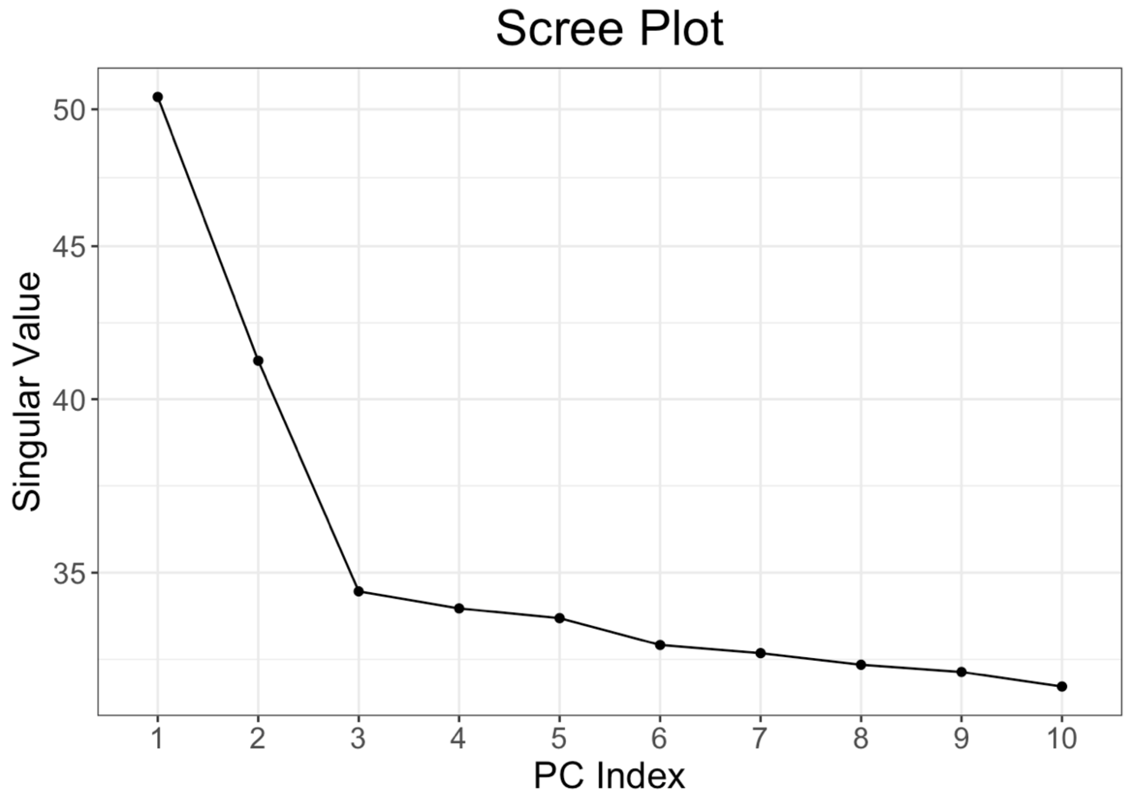

Supp. Figure S4: WECARE-Group1 + NFFE PCA Scree plot

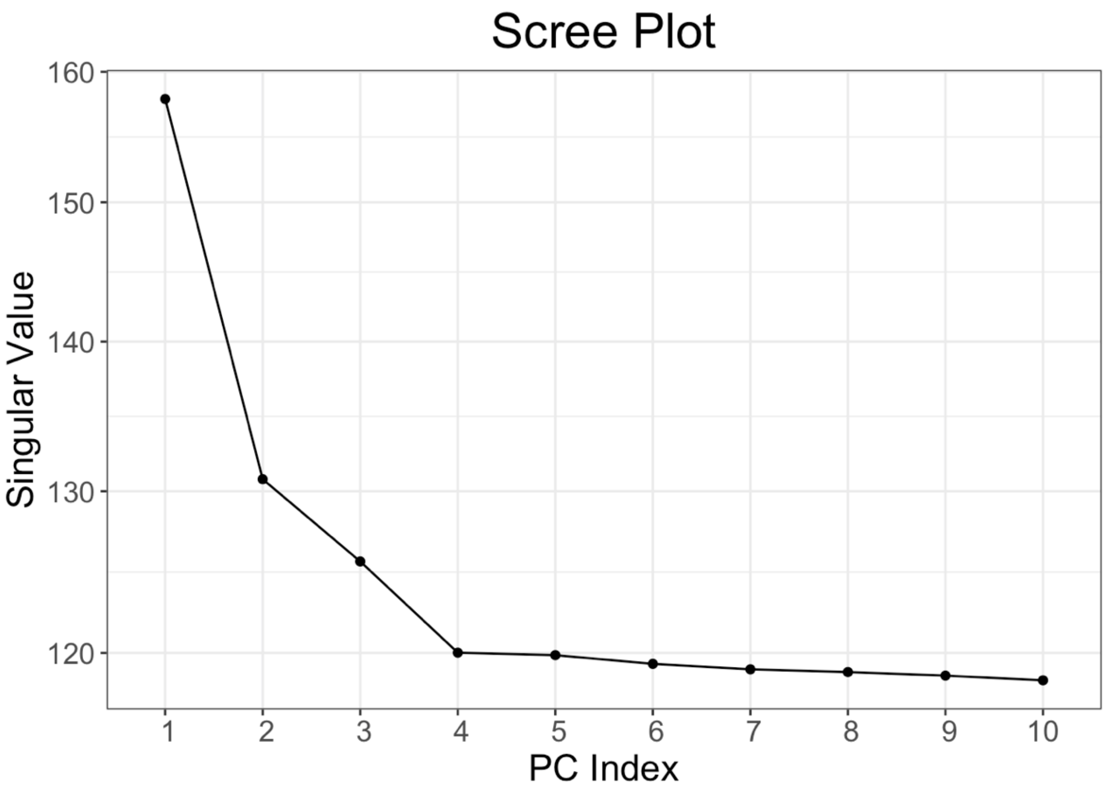

Supp. Figure S5: Burden of PGVs stratified by latency

A: Group 1

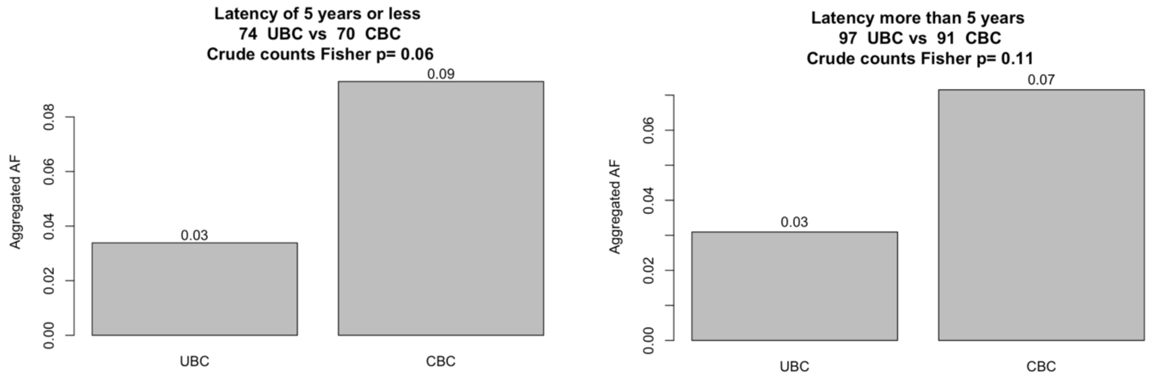

B: Group 2

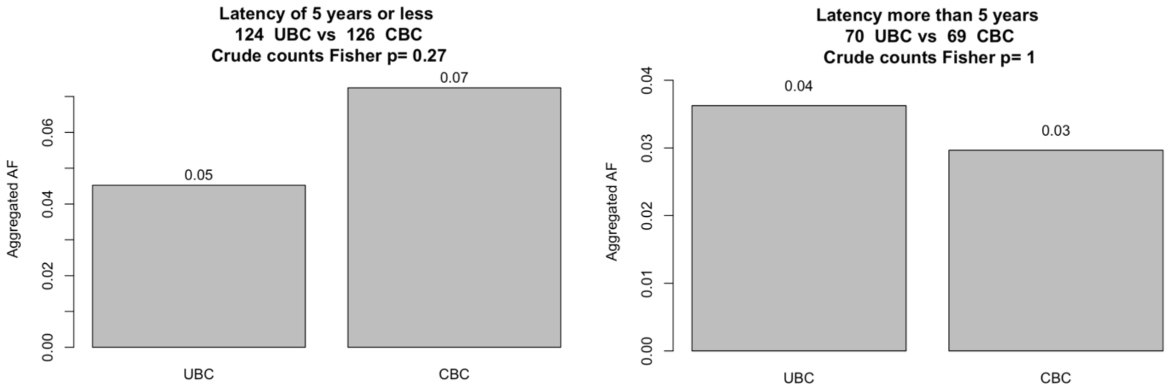

Note: In CBC the latency is the time between the 1st cancer and 2nd cancer (lack of 2nd cancer in UBC)

Supp. Figure S6: WECARE-Group1 + NFFE PGVs burden

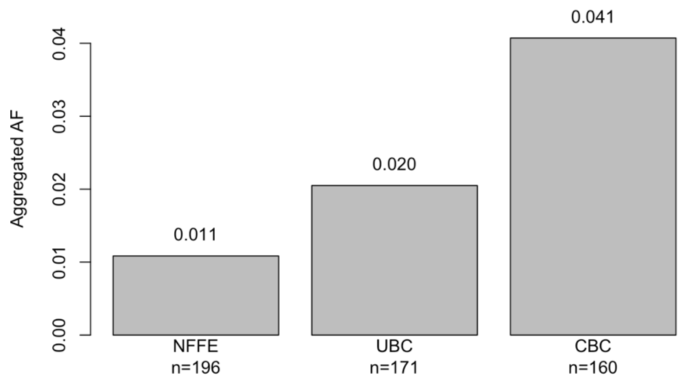

Note: The NFFE<UBC<CBC trend is statistically significant (POLR test p = 0.02). The absolute AF values are smaller than in WECARE-only analysis because variants with low coverage in NFFE samples were removed (see Figure 1 and Methods section in the main text).
